# Atomically-resolved interlayer charge ordering and its interplay with superconductivity in YBa_2_Cu_3_O_6.81_

**DOI:** 10.1038/s41467-021-24003-0

**Published:** 2021-06-23

**Authors:** Chun-Chih Hsu, Bo-Chao Huang, Michael Schnedler, Ming-Yu Lai, Yuh-Lin Wang, Rafal E. Dunin-Borkowski, Chia-Seng Chang, Ting-Kuo Lee, Philipp Ebert, Ya-Ping Chiu

**Affiliations:** 1 Department of Physics, National Taiwan University, Taipei, Taiwan; 2 Institute of Atomic and Molecular Sciences, Academia Sinica, Taipei, Taiwan; 3 Peter Grünberg Institut, Forschungszentrum Jülich GmbH, Jülich, Germany; 4 Institute of Physics, Academia Sinica, Taipei, Taiwan; 5 Department of Physics, National Sun Yat-sen University, Kaohsiung, Taiwan; 6 Center of Atomic Initiative for New Materials, National Taiwan University, Taipei, Taiwan

**Keywords:** Superconducting properties and materials, Characterization and analytical techniques

## Abstract

High-temperature superconductive (SC) cuprates exhibit not only a SC phase, but also competing orders, suppressing superconductivity. Charge order (CO) has been recognized as an important competing order, but its microscopic spatial interplay with SC phase as well as the interlayer coupling in CO and SC phases remain elusive, despite being essential for understanding the physical mechanisms of competing orders and hence superconductivity. Here we report the achievement of direct real-space imaging with atomic-scale resolution of cryogenically cleaved YBa_2_Cu_3_O_6.81_ using cross-sectional scanning tunneling microscopy/spectroscopy. CO nanodomains are found embedded in the SC phase with a proximity-like boundary region characterized by mutual suppression of CO and superconductivity. Furthermore, SC coherence as well as CO occur on both CuO chain and plane layers, revealing carrier transport and density of states mixing between layers. The CO antiphase correlation along the **c** direction suggests a dominance of Coulomb repulsion over Josephson tunneling between adjacent layers.

## Abstract (web summary)

Charge ordering and superconductivity are known to compete in layered cuprates; however, precise real-space characterization of their interplay has been lacking. Here, the authors address this using atomically-resolved cross-sectional scanning tunnelling microscopy and spectroscopy on cryogenically cleaved YBa_2_Cu_3_O_6_._81_.

## Introduction

Charge ordering in layered cuprates has been identified as leading competing order to superconductivity and hence as a key factor in shaping and defining the phase diagram^1^. Thus, elucidating the nanostructure of charge order (CO) and its spatial interplay with superconductivity are particularly important for understanding high-temperature superconductivity in cuprates^2–11^. Therefore, CO and its nanostructure have attracted considerable interest for explaining various phenomena: For example, the spatial configuration of CO has been investigated by several X-ray measurements, which led to the discovery of anti-phase interlayer correlations of the charge density wave (CDW) in YBa_2_Cu_3_O_6+*x*_ (YBCO_6+*x*_)^12,13^ and a three-dimensional (3D) CDW in thin YBCO_6+*x*_ films^14^. The existence of 3D CDWs suggests that charge reservoir layers may act as bridges to transmit a CO correlation along the **c** axis of cuprates. However, the exact role of charge reservoir layers in the formation of CO is still unclear and under debate.

Resolving these critical physical issues and thereby generating a fundamental description of the spatial interplay of CO and superconductivity in YBCO_6+*x*_, requires an atomically resolved real-space characterization of the electronic structure and density-wave correlation layer-by-layer along the **c** axis. Unfortunately, such results are lacking, despite the prospect to contribute centrally to a fundamental understanding of the competing orders of CDW and superconductivity (SC).

Scanning tunneling microscopy (STM) and spectroscopy (STS) are ideally suited to provide a direct atomically resolved access in real space to electronic properties and thus promises a direct insight into the nature of CO in YBCO_6+*x*_. Prior STM experiments have demonstrated the success of detecting the atomic and electronic structure in real-space on the cleaved **c** (001) planes^15,16^. Yet, in these measurements, no CuO_2_ plane can be probed, due to cleavage in between of BaO or CuO-chain layers^15^. To overcome this restriction, on the one hand, (100) oriented (**a**) growth surfaces of YBCO_6+*x*_ were probed in situ^17,18^, and on the other hand, cross-sectional STM (XSTM) measurements^19,20^ were done on fractured surfaces, albeit without atomic resolution.

In this work, we use a low-temperature cleavage stage to cleave atomically flat cross-sectional surfaces of highly-doped YBa_2_Cu_3_O_6.81_ (*p* $\cong$ 0.14)^21^ layers (room temperature cleavages led to unstable fractures only). The cryogenic cleavage reveals that CO nanodomains are embedded in the SC phase, with a proximity-like boundary region characterized by mutual suppression of CO and superconductivity. Furthermore, Cooper pair coherence in the SC phase, as well as charge ordering in the CO phase, occur on both CuO chain and plane layers, indicating carrier transport and density of states mixing between the layers. The antiphase CO correlation along the **c** direction suggests the dominance of Coulomb-interactions between adjacent layers over the Josephson tunneling. Our atomically resolved observations directly reveal the 3D nature of charge order in YBCO, and shed light on the importance of the role played by oxygens for interlayer coupling and complex orders as well as their competition in cuprates^22–24^.

## Results

**Fig. 1:**
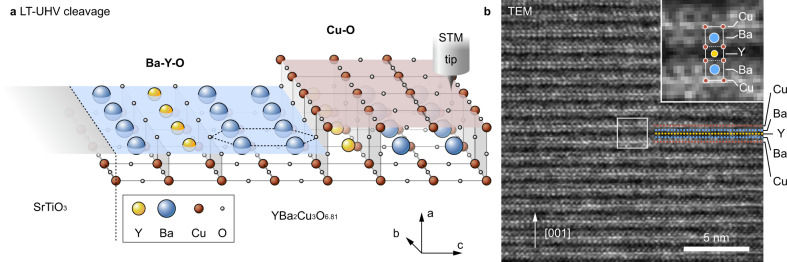
YBCO cleavage surfaces and layered structure. **a** Schematic illustration of cross-sectional STM measurements on the YBa_2_Cu_3_O_6.81_/SrTiO_3_ cleavage surfaces, which are either Ba–Y–O or Cu–O terminated. **b** Bright field cross-sectional TEM image (200 keV) taken 50 nm away from the YBa_2_Cu_3_O_6.81_/SrTiO_3_ interface revealing the epitaxial YBCO layers. The inset shows a magnification with an indicated unit cell.

**Fig. 2:**
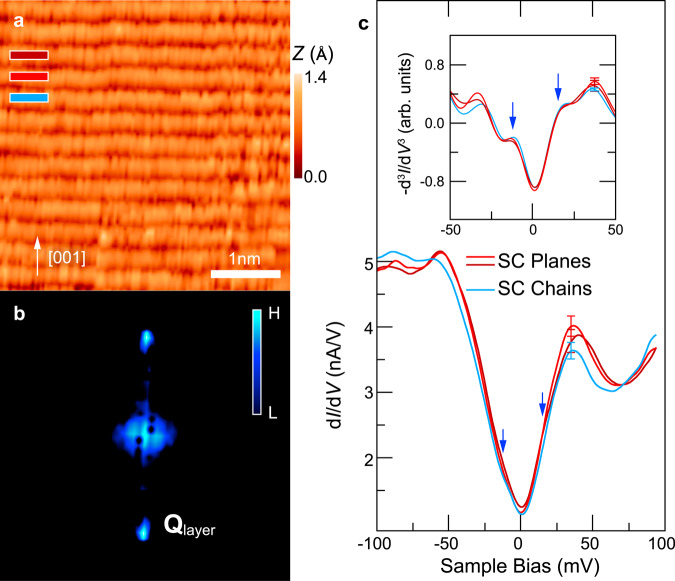
Superconducting region on Cu–O terminated surface. **a** Constant-current STM image of the Cu–O terminated YBCO surface measured at a sample bias of −50 meV and a current of 200 pA. The STM image exhibits a clear layered structure perpendicular to [001] direction, corresponding to tunneling into states associated with the CuO-chain and CuO_2_-plane layers. **b** Fourier transform of the STM image in (**a**). The peaks labeled **Q**_layer_ arise from the imaged atomic planes. **c** Differential conductance spectra obtained from three consecutive atomic layers corresponding to CuO-chain and CuO_2_-plane layers. The blue arrow marks the superconductive (SC) gap as a weak bulge on the flanks of the pseudogap. The inset displays the negative second derivative of the differential conductance spectra, −d^3^*I*/d*V*^3^, measured from the three consecutive layers, revealing the coherence peaks of the SC gap and providing the signature to identify the SC phase. A representative error bar is given for each spectrum.

Due to the bulk structure of orthorhombic YBCO, ideally cleaved (100) surfaces are terminated either by Ba–Y–O or by Cu–O layers^19^ (see Fig. 1a). In our XSTM measurement, we indeed observe two types of cleavage surfaces: The former one exhibits a stretched hexagonal-like pattern compatible with a Ba–Y–O terminated surface (see Supplementary Note 1). The latter one, whose STM image is illustrated in Fig. 2a, is characterized by atomic rows perpendicular to the (001) **c** direction with a periodicity of 0.36 nm. Based on the distinct electronic structure of each layer in YBCO, it can be deduced that the bright rows with higher tunneling conductance arise primarily from copper atoms, which contribute most of the local density of states (LDOS) at the used sample bias of −50 mV^25^. The dark depressions in between, with lower tunneling conductance, correspond to the insulating layers with barium and yttrium atoms, which do not contribute to the tunnel current significantly. Interestingly, CuO chain and CuO_2_ plane layers show an identical contrast in STM images. Figure 2b illustrates the Fourier transform image of Fig. 2a. Only a bright peak labeled **Q**_layer_ is resolved, which corresponds to the spacing between the atomic rows (i.e., the CuO layers) of 0.36 nm.

Furthermore, we acquired tunneling spectra on the CuO-terminated surface on top of the atomic rows (Fig. 2c). Since the crystal structure of YBCO consists of two CuO_2_ planes separated by one CuO chain layer along the **c** direction, we average the spectra acquired on every third atomic row, as marked in Fig. 2a, in order to identify the CuO chain layers. The spectra of all layers reveal superconductive properties: The differential conductance (d*I*/d*V*) spectra exhibit a pronounced v-shaped dip structure, which is the signature of a pseudogap, ranging approximately from −50 mV to +46 mV, in line with the SC critical temperature *T*_C_ = 85 K. Superimposed on and thus somewhat hidden in the intense pseudogap slopes one can extract in addition the existence of coherence peaks^26^ and hence a superconductive gap using the negative second derivative of the differential conductance (i.e., −d^2^/d*V*^2^ (d*I*/d*V*) = −d^3^*I*/d*V*^3^) (inset), which is known to be a sensitive indicator of the position of coherence peak^27^ (since its maxima amplify peaks in the conductance by removing linear backgrounds). Coherence peaks are present in spectra of all layers, suggesting the presence of superconductivity in both CuO_2_ plane and CuO chain layers. The SC gap is estimated to be 15 ± 2 mV. Note, the apparently weak SC coherence peaks could be related also to the fact that the spectra are acquired in a plane involving the **c** axis, along which the SC coherence length is expected to be shorter.

Despite the similarity, a subtle difference can be observed in one of the spectra in Fig. 2c: the blue spectrum exhibits suppressed pseudogap peaks (at negative voltage almost disappearing) as compared to the red and dark red spectra, which are almost identical. Taking into account the crystallography of YBCO, one can assign the blue colored spectrum to the CuO chain layers, and the red/dark red colored ones to the CuO_2_ plane layers.

**Fig. 3:**
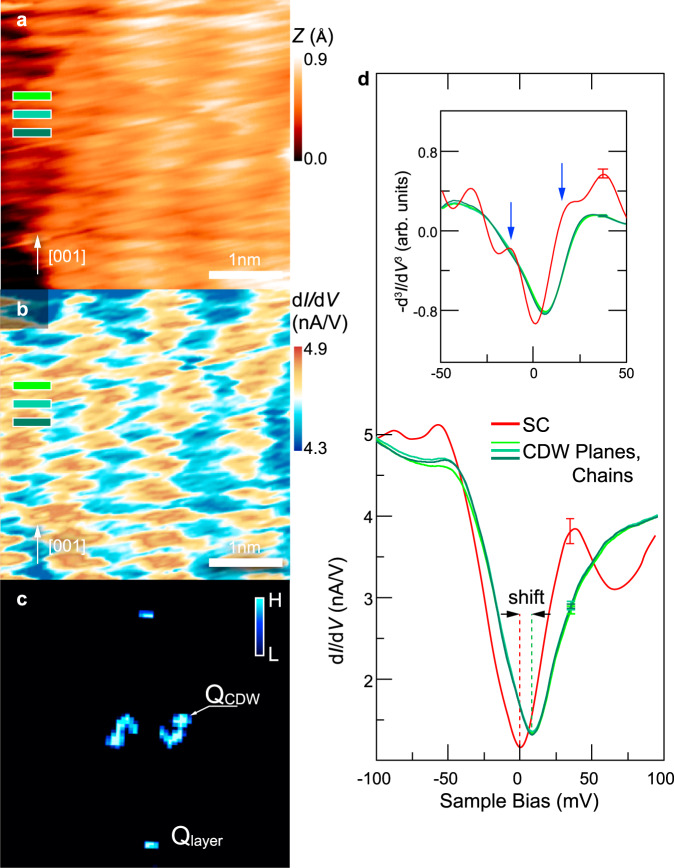
Charge density wave (CDW) region on Cu–O terminated surface. **a** Topographic image obtained with a sample bias of +50 mV and 200 pA, showing the presence of the charge modulation along the **b**-axis. **b** The corresponding differential tunneling conductance map at +50 mV sample bias. **c** Fourier transformation of the image in (**b**). **d** Differential tunneling spectra in CDW region obtained with a setpoint of −50 mV and 200 pA. The red spectrum shows the average LDOS in SC-phase for comparison. Spectra colored in different shades of green correspond to d*I/*d*V* taken from three consecutive layers. The inset shows −d^3^*I*/d*V*^3^, in which the superconducting gap is marked by the blue arrows. A representative error bar is given for each spectrum.

In addition to the dominant SC phase presented above, a minority CO phase occurs in about 15% of the investigated surface area. Figure 3a displays a STM image of a CO phase, which is characterized by height modulations along the atomic rows at the Cu–O terminated surface. The differential conductance image (Fig. 3b) of the same area reveals a charge modulation or CDW as origin of the height modulation. The CDWs are hole density modulations in a hole-doped Mott insulator^7^ and can be considered to be the real space signature of the CO phase. The CDW’s wavelength of 1.2 nm shows up in the Fourier transform (Fig. 3c) as additional (twin) peak (labeled **Q**_CDW_). The position of the atomic row-induced peak **Q**_layer_ remains fixed, corroborating the interpretation that the CO-induced modulation is superimposed on the contrast of the atomic rows. The appearance of **Q**_CDW_ twin peaks is related to the anti-phase correlation of CO along the **c** direction discussed below.

Furthermore, Fig. 3d shows the averaged d*I*/d*V* spectra measured on three consecutive layers in the CO area (green lines). For comparison an average d*I*/d*V* spectrum of the SC phase area is shown as red line (average of spectra in Fig. 2c). Four distinct features can be recognized: (i) no significant difference is observed between CuO chain and CuO_2_ plane layers in CO area. (ii) The d*I*/d*V* curves in the CO domain reveal strongly suppressed pseudogap peaks (at −50 meV and +46 meV) as well as no local dip at +65 meV. (iii) The −d^3^*I*/d*V*^3^ spectra reveal no SC coherence peaks. (iv) Finally, a clear upward shift by 7 meV of the pseudogap peak and the gap’s minimum occurs, as compared to the SC region. These features demonstrate that the CO phase is distinctively different than the SC phase. In particular, we note that no CO can be detected in the SC phase as supported by the different electronic properties and the absence of any modulation in constant current as well as conductivity maps (Fig. 2a and Supplementary Fig. 2). Note, these results rule out quasiparticle scattering as origin of the observed modulations, since quasiparticle scattering should only occur in presence of SC^11^. Hence, the dominant contributions to the modulations observed in STM can be assigned to CDWs.

The most intriguing LDOS feature in the CO domain is the upward shift of the pseudogap minimum, which suggests a particle-hole asymmetric gap. The shift creates a significant increase of the density of states (DOS) directly at the Fermi level (*E*_F_), which is in conflict with a strongly reduced single-particle DOS at *E*_F_ in the SC phase. Hence, we anticipate that the shift of the minimum of single-particle DOS away from *E*_F_ is intimately interrelated with and sets the basis for CO^28,29^. As side effect, the upward shift can be anticipated to result in reducing the Cooper pair bonding energy and hence the Cooper pair concentration and coherence, thereby suppressing superconductivity.

**Fig. 4:**
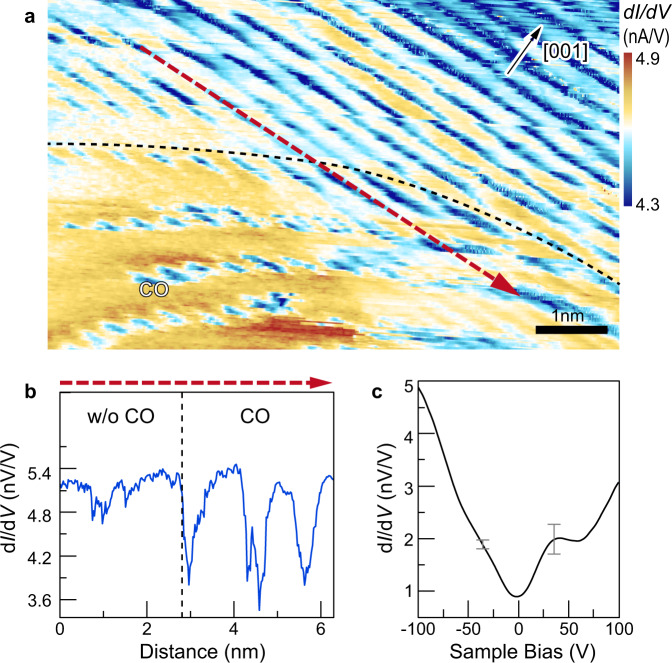
The CO phase boundary and STS at the boundary. **a** Differential conductance map of the CO phase boundary measured at a sample bias of +200 mV and tunneling current of 500 pA. The dashed line indicates the approximate position of the CO phase boundary. **b** We used line profiles along the atomic rows in **b** direction to define the CO phase boundary. The CO phase shows up as a wavy corrugation (height change), which suddenly terminates. The spatial position where the modulation disappears and the contrast becomes constant is taken as boundary. **c** Average tunneling spectrum measured at the CO phase boundary with two representative error bars given at positive and negative bias.

Furthermore, Fig. 4a and Supplementary Fig. 3 illustrates that nanoscale CO domains^30,31^ are coexisting with and inserted in between of SC areas. Figure 4b illustrates the average tunneling spectrum measured at the boundary. It exhibits features, which distinguish it from both the spectra measured in the center of the CO domain and in the SC phase: first, the minimum of the DOS dip is again at the Fermi energy and a pseudogap is formed, although with weakly developed edge peaks. Second, no superconductive energy gap and coherence peaks can be detected. Hence, a mutual suppression of CO and SC occurs in a proximity-like boundary region.

**Fig. 5:**
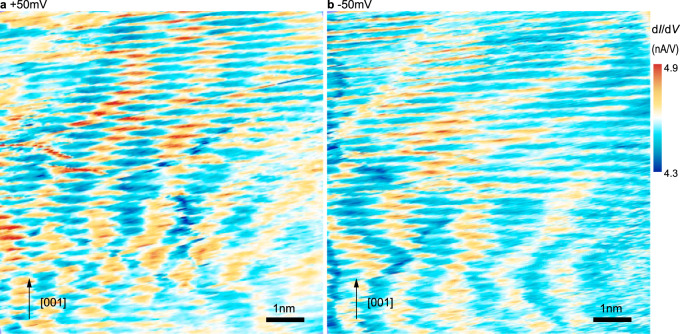
Dependency of CDW pattern on the polarity of sample bias. **a**, **b** Conductance map of a surface area within a CO domain measured at sample bias of **a** +50 mV and **b** −50 mV. The tunnel current is 200 pA.

**Fig. 6:**
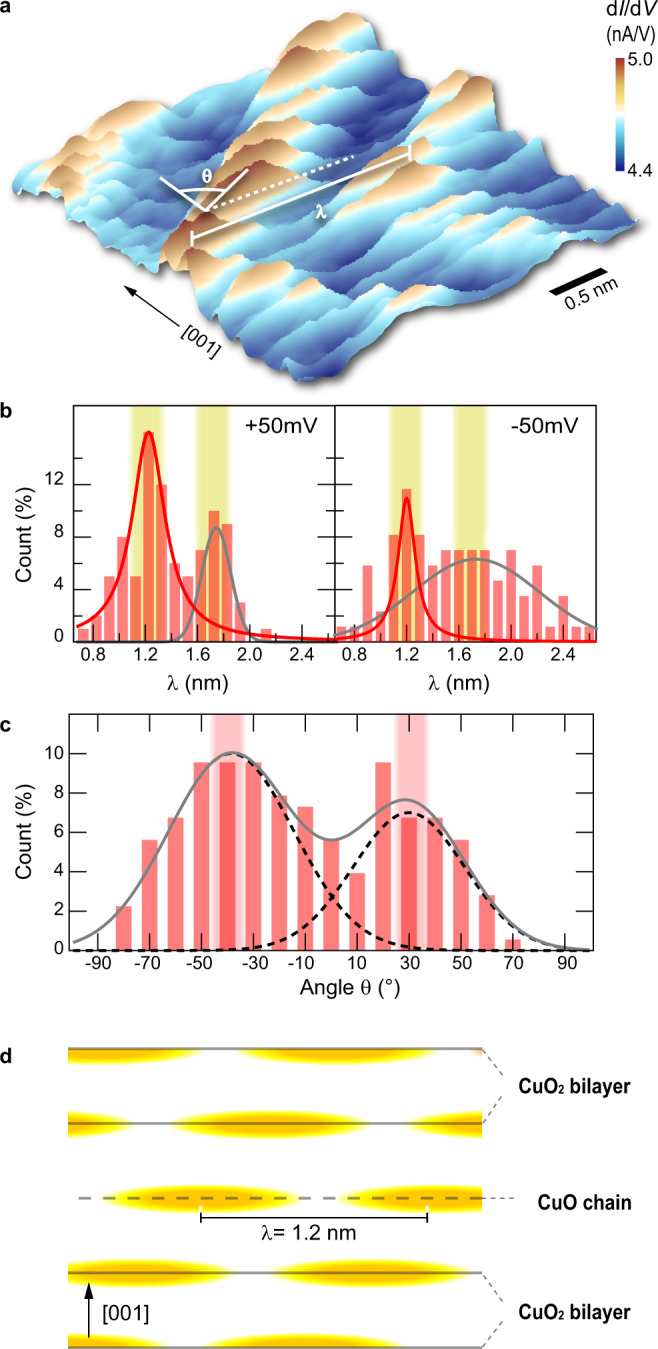
Wavelength and angular distribution of CDW. **a** Three-dimensional representation of a d*I*/d*V* image showing charge modulations on the atomic rows in a false-color scale (acquired at a tunnel voltage of +50 mV). Indicated are the definitions used to measure the wavelength *λ* and the angle *θ* describing the spatial correlation of charge modulations in adjacent atomic rows. **b** Histograms of the distribution of wavelength *λ* measured at (left) +50 mV and (right) −50 mV tunneling voltage. One can distinguish a peak at *λ* = 1.2 nm (red lines: Lorentzian fits) and another peak at ~*λ* = 1.6 nm (gray lines are Gaussian fits) for both tunneling voltages. **c** Frequency distribution of the angle θ describing the correlation of the charge modulation across the atomic rows in **c** ([001]) direction. Angles *θ* can be best described with two Gaussians, centered around −40° and +30°. The presence of two shifted peaks indicate a short-range anti-correlation ordering of parallel CDW stripes along the [001] direction, as schematically illustrated in (**d**).

After the electronic characteristics of CO and SC phases as well as their boundary, we address the real space pattern of the CDWs in Figs. 5 and 6. Since it is known that the intensity of the wave vector and thus the real space modulation intensity of the CDW reaches a maximum at the pseudogap energy^11^ (i.e., ±50 meV) we chose these voltages for mapping the modulations of the CO. Figure 5a and b illustrate a pair of d*I*/d*V* maps probed at +50 and −50 mV, revealing CDW-induced modulations. The two wave patterns are not identical, suggesting a voltage dependence of the charge modulation^16,32^. For a deeper understanding of the CDW, we evaluate its wavelength *λ* (i.e., the peak-to-peak distance between two modulation maxima as defined in Fig. 6a) for every atomic row (i.e., chain and plane layers) in all of the sampled CO region. Figure 6b shows the obtained distributions of *λ* at bias voltages of +50 and −50 mV, respectively. Both distributions exhibit two peaks centered around 1.2 and 1.6 nm wavelength. The data suggest that the dominating wavelength shifts from 1.2 nm at +50 mV to 1.6 nm at −50 mV. Furthermore, one can discern a general curvy pattern of the CDW maxima near the edges of the nanoscale CO domains (Fig. 4a), where the longer wavelength is preferentially present. For understanding the distribution of wavelength, we recall that the periodicity of CO depends on the doping level of YBCO^2^. Thus, for a given doping level, the periodicity of CO is an intrinsic property. The wavelength of *λ* = 1.2 nm is consistent with the position of the CDW’s Fourier peak in Fig. 3c and with the wave vectors of CDWs measured by X-ray experiments^2,9^, coinciding with but not necessarily originating from the oxygen-site occupation induced periodicity of chain superlattice in ortho-III ordered YBCO^33^. The presence of longer wavelengths can be explained on basis of fluctuations of the local oxygen site occupation, especially near the CO phase boundary. Note, due to the possible influences of the tip’s electric field on the sample and the curvy pattern especially near the edges of the CO phase areas, one cannot derive an energy-dependent dispersion from the measured voltage dependence.

Besides the presence of charge-order modulation on single layers, the STM images indicates a correlation of the CDWs along the perpendicular **c** ([001]) direction. In order to support the correlation, we evaluate the angle *θ* defined in Fig. 6a as the angle between the **c** direction and the stripe-like CDW pattern. Figure 6c illustrates the obtained distribution of occurrences of angles *θ*, which can be best described with two Gaussians, centered around −40° and +30°. Near 0° a minimum occurs. The distribution significantly deviates from that expected for uncorrelated CDWs (random distribution) or for in phase CO correlation in [001] direction (single peaks centered at 0°). The presence of two shifted peaks indicates a phase-shifted interlayer CO correlation in [001] direction, i.e., a short-range anti-correlation ordering of parallel CDW stripes along the [001] direction, as schematically illustrated in Fig. 6d.

## Discussion

The above results raise several points, which require an in-depth discussion: (i) the relation of CO and SC phase, (ii) the layer resolved SC properties, (iii) the layer resolved CO, and (iv) the anti-phase CO correlation across the layers.

First, the observation of CO domain embedded in the SC phase can be rationalized as follows: The periodicity of the CO is known to depend directly on the oxygen content (i.e., hole concentration^2,34^). This suggests that the samples exhibit local fluctuations of the hole concentration^30^ in analogy to dopant fluctuations in semiconductors^35^. These fluctuations can be expected to induce local CO domains with hole density changes toward the edges of the domain, giving rise to curvy CO pattern. Hence, the formation of CO nanoscale domains can be understood in terms of quenched disorder and local inhomogeneity in cuprate superconductors.

With this in mind, we anticipate that the electronic features at the CO–SC-phase boundary reflect a reduced free carrier concentration at *E*_F_ as compared to that in the center of the CO domain. This reduced carrier density suppresses CO and allows Cooper pairs to form, which can be inferred from the reformation of a pseudogap with edge peaks. However, no SC coherence peaks are detected in the boundary region. One could call this a proximity effect between CO and SC phases, in analogy to SC-metal contacts. This suggests that CO and SC are indeed competing and immiscible orders. This is corroborated by the fact that although both phases occur in the same sample, the respective electronic features and order parameters (SC coherence peaks and shift of DOS minimum) do not overlap and thus the phases are not found to intermix. The tunneling spectra suggest that the competition is likely defined by the competition for the charge carriers, as both phases need carriers, one for a superconducting state, and the other as free single carriers which undergo a CO. Note, at present there is not experimental data on how wide the boundary region is. Furthermore, defects in the YBCO stacking order should not be responsible for the formation of CO phase embedded in SC phase, because the size of areas free of stacking defects is significantly larger than the observed size of the CO areas.

Second, in the SC phase, we observed that the d*I*/d*V* curves of the CuO chain and CuO_2_ plane layers exhibits the same overall shape including a superconducting gap and the corresponding coherence peaks (Fig. 2). This suggests that both, the chain and plane layers, exhibit superconductive properties, in line with previous observations of a SC gap in the chain layer^36,37^. This and the observation of a SC gap in tunneling spectra of the Ba–Y–O terminated surface (see Supplementary Fig. 1) indicate that electrons can tunnel/hop from layer to layer in YBCO, due to the small layer separation of about 0.4 nm. Similarly, we anticipate that Cooper pairs are able to cross insulating layers via Josephson tunneling. In addition, one can speculate that the oxygen atoms in insulating Ba–O layers and their bonds provide a channel for carriers and Cooper pairs to connect neighboring CuO chain/plane layers. As a consequence, one would expect to observe the same single-particle DOS on both chain and plane layers in terms of SC gap and coherence peaks, decaying along the **c** direction, but with significant overlap between adjacent Cu–O layer. This picture of carrier and Cooper pair hopping/tunneling is directly supported by our measurements (see also Supplementary Note 4) and could be related to previously suggested possible intermixing of the LDOS between CuO chain and CuO_2_ plane layers^36,37^.

Third, we address the presence of CO in CuO chain and plane layers and its correlation across the layers. These findings confirm again the possibility of carrier hopping/tunneling between the Cu–O layers and indicate an interaction of the CO across the CuO layers. The presence of an interaction of CO across the CuO layers can be also inferred from a prior observed transition of a 3D-like to a 2D-like CO behavior with decreasing doping, i.e., increasing oxygen vacancy concentration^14^. In the highly underdoped YBCO with unoccupied oxygen sites in the CuO chain layers, the copper atoms (in the chain layer) have essentially a fully occupied 3*d*^10^ electron configuration. As the oxygen content increases, the vacancy sites in the chain are occupied by oxygen and the coppers’ electron configuration evolves from 3*d*^10^ toward 3*d*^9^. This results in holes located at the oxygen atoms in the CuO chain. Hence, for strongly underdoped YBCO, the Cu atoms in the chain layer exhibit a fully occupied 3*d* shell, which one can anticipate to block the electron pathway between the adjacent layers in **c** direction resulting in a 2D-like behavior. In contrast, in optimally doped YBCO, the Cu atoms in the chain layer have an incompletely filled 3*d* shell, which should allow electron hopping between the layers, resulting in a 3D-like electronic behavior. The presence of such conduction pathways is further corroborated by an electronic rehybridization of the Cu(chain)-apical O(4)-Cu(plane)-orbitals^23^.

Finally, we turn to the anti-phase correlation of CO across the layers (showing up in the angle distribution in Fig. 6c and schematically illustrated in Fig. 6d). The CO correlation across the layers has been suggested to be governed by Josephson tunneling as well as charge interaction between the layers^38^. Since Josephson tunneling favors an in-phase correlation, our experimental results suggest that the antiphase pattern observed here is dominated by the minimization of Coulomb interaction energy between layers^5,38^. As further support for the 3D CO structure, we recall that on the Ba–Y–O terminated surface we never observed any charge density modulation, suggesting that indeed no CO occurs on the Ba–Y–O layer. This corroborates the identification of the CO consisting of one-dimensional stripes along the **b** direction on the Cu–O planes with anticorrelation in **c** direction.

We revealed the interlayer coupling and the spatial interplay and competition of the charge ordered and the superconductive phases in YBa_2_Cu_3_O_6.81_ by direct real-space imaging with atomic-scale resolution using cross-sectional scanning tunneling microscopy and spectroscopy on cryogenic cleavage surfaces. We found that highly-doped YBCO with high *T*_C_ contains nanosized 3D-correlated CO domains embedded in the SC phase, and attribute this to fluctuations of the local doping. CO is found to be antiphase correlated in **c** direction suggesting a dominance of Coulomb-repulsion over Josephson-tunneling. In the SC phase the tunneling spectra reveal the presence of a SC energy gap and coherence peaks on CuO chain as well as plane layers. In the CO phase, an upward shift of the minimum of the pseudogap, attributed to an asymmetric particle hole gap, and no coherence peaks were found on both CuO chain and plane layers, too. The presence of almost identical LDOS on chain and plane layers in both phases is believed to be a manifestation of carrier transport across the CuO layers and indicative of a density of states mixing, anticipated to be driven by the rehybridization of apical oxygen atoms with the neighboring Cu atoms in the chain and plane layers. At the CO–SC phase boundary, the tunneling spectra reveal a proximity-like zone, where neither SC gap nor the upward DOS shift characteristic of the CO phase, can be detected, suggesting a mutual suppression of CO and superconductivity. We anticipate that both phases are competing for the same carriers and are thus immiscible orders. The results revealed by cross-sectional STM/STS provide direct evidence of the 3D nanostructure and spatial interplay of competing, immiscible orders with superconductivity and a fundamental insight into the role of apical oxygen atoms for interlayer coupling and understanding complex competing orders in cuprates.

## Methods

For the experiments we investigated a 400 nm thick (001) oriented YBCO_6.81_ (YBCO) thin film, grown on a SrTiO_3_ (STO) with (001) oriented substrate using pulsed laser sputter deposition. Pieces cut from the sample were cleaved on cross-sectional a (100) planes at 10 K in-situ in the ultrahigh vacuum (UHV) chamber. Immediately after cleavage the samples were investigated at 4.3 K by STM/STS without interruption of the vacuum after cleavage. Figure 1a shows a schematic illustration of the bulk-like cleavage planes and the used low-temperature XSTM setup. The low-temperature (LT) cleavage and measurement avoids changes of the oxygen content under UHV conditions, essential for probing intrinsic properties of YBCO on bulk-like cleavage surfaces. Furthermore, in order to explore unstrained YBCO, we probed a region about 150 nm away from the YBCO/STO interface, where the stress from the lattice mismatch is generally released. Finally, throughout the YBCO film we observed a well-ordered **c** axis oriented atomic structure, as illustrated by the bright field transmission electron microscopy (TEM) image in Fig. 1b. The YBCO had a high critical temperature of *T*_C_ ≅ 85 K.

STS measurements were carried out using the lock-in technique (bias modulation δ*V* = 2 mV, *f* = 708 Hz). Using this lock-in technique, standard d*I*/d*V* vs. *V* spectra as, well as topographic differential conductance maps d*I*/d*V*(**r**, *V*_set_) ≡ g(**r**, *V*_set_) for a fixed set voltage *V*_set_, were acquired. The latter is used to map across the surface the LDOS information at the energy corresponding to the set voltage.

For obtaining a tunneling spectrum at a selected pixel point, i.e., spatial positions, five d*I*/d*V* spectra were measured consecutively and averaged. Then, these tunneling spectra were further averaged for spatially equivalent pixel positions, e.g., along the same type of atomic plane/row. Finally, a running average with a width of 3 mV was applied for smoothing, resulting in the averaged spectra shown in Figs. 2c, 3d, and 4c.

## Supplementary information

Supplementary Information

## Data Availability

The data that support the findings of this study are available from the corresponding author upon reasonable request.
