# Supplementary material for: Atomically-resolved interlayer charge ordering and its interplay with superconductivity in YBa2Cu3O6.81
Source: Nat Commun. 2021 Jun 23;12:3893. doi: 10.1038/s41467-021-24003-0 (PMC8222377; doi:10.1038/s41467-021-24003-0)
Supplement: Supplementary file 1 — Supplementary Information [file 41467_2021_24003_MOESM1_ESM.pdf]

Supplementary Information

Atomically-resolved interlayer charge ordering and its interplay with superconductivity in $YBa_2Cu_3O_{6.81}$

Chun-Chih Hsu[1], Bo-Chao Huang[2], Michael Schnedler[3], Ming-Yu Lai[2], Yuh-Lin Wang[1,2], Rafal E. Dunin-Borkowski[3], Chia-Seng Chang[4], Ting-Kuo Lee[5], Philipp Ebert[3], and Ya-Ping Chiu[1,4,6]

[1]*Department of Physics, National Taiwan University, Taipei 10617, Taiwan*

[2]*Institute of Atomic and Molecular Sciences, Academia Sinica, Taipei 10617, Taiwan*

[3] *Peter Grünberg Institut, Forschungszentrum Jülich GmbH, 52425 Jülich, Germany*

[4] *Institute of Physics, Academia Sinica, Taipei 11529, Taiwan*

[5] *Department of Physics, National Sun Yat-sen University, Kaohsiung 80424, Taiwan*

[6] *Center of Atomic Initiative for New Materials, National Taiwan University, Taipei 10617, Taiwan*

1. Atomically-resolved Ba-O terminated surface

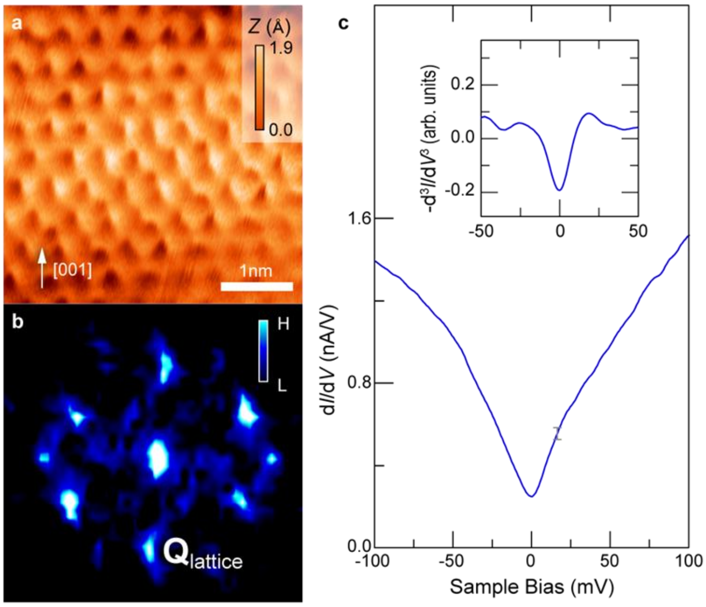

Supplementary Fig. 1: a Constant-current STM image of Ba-Y-O terminated surface acquired at a sample bias of -200 mV and tunneling current of 60 pA. **b** The Fourier transform of (a), revealing apparently hexagonally arranged peaks. The structure arises from imaging the LDOS at the Ba and Cu atoms. Y atoms do not contribute significantly at the used voltage. This leads to the particular atomic scale corrugation arrangement. **c** Average d*I*/d*V* spectrum obtained on **a**. The inset displays the negative of the second derivative of differential conductance spectrum $-d^3I/dV^3$. The peaks are attributed to the SC gap. A representative error bar is given in the spectrum.

In our cross-sectional scanning tunneling microscopy study, two types of cleaved surface are found. They arise from a cleavage between two different atomic *a*-plane layers in the ideal $YBa_2Cu_3O_{6.81}$ (YBCO) structure: One is a Ba-Y-O terminated and the other one is a Cu-O terminated surface. Supplementary Fig. 1 shows the STM and STS data acquired on the Ba-Y-O-terminated cleavage surface. In the constant-current STM images (Supplementary Fig. 1**a**) the Ba-Y-O-terminated surface exhibits a stretched hexagonal-like pattern, which is more vividly seen in the Fourier transform image (Supplementary Fig. 1**b**). This pattern in the STM images arise from the imaging of the local density of states (LDOS) localized primarily

at the Ba and Cu atom position, since the LDOS contribution of Y is negligible at the used tunneling voltage[21]. Supplementary Fig. 1**c** illustrates the average tunneling spectra on Ba-Y-O surface. The inset of Supplementary Fig. 1**c** shows the negative of the second derivative of tunneling spectra $-d^3I/dV^3$, revealing two peaks that coincide with superconductive (SC) coherence peaks. This observation indicates that Cooper pairs on Cu-O layers tunnel through the Ba-Y-O layers.

The observation of a SC gap with coherence peaks on the Ba-Y-O terminated surface implies that electrons are able to tunnel from one to the next layer in YBCO. Due to the insulating properties of the top Ba-Y-O surface layer, the surface layer has a wider gap than the underlying superconductive Cu-O layer. Thus, one can anticipate that the electrons tunnel from the Cu-O layer through the Ba-Y-O layer into the tip or vice versa. As a result, the underlying single particle density of states is probed, just the effective tip-sample distance is increased by the interlayer separation of 0.19 nm. The possibility of tunneling also suggests that Cooper pairs are able to cross-insulating layers, as the separation between CuO chain/plane layers is only about 0.4 nm.

2. The absence of CO in the SC region

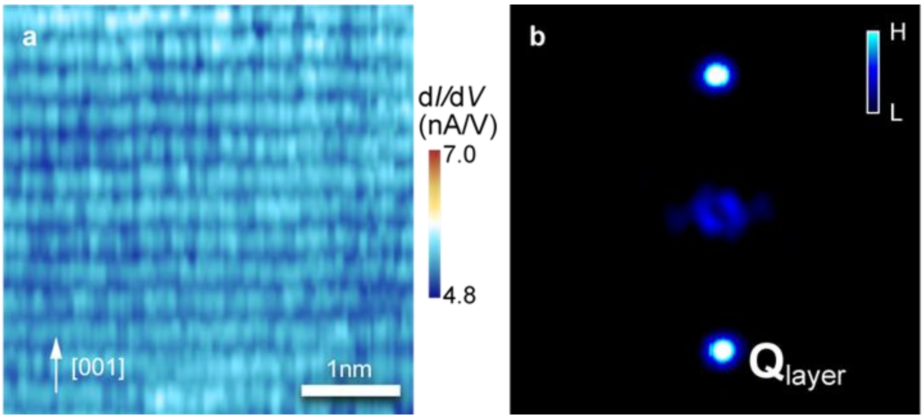

Supplementary Fig. 2: **a** Differential conductance map (V = -50 mV) and **b** corresponding Fourier image of the differential conductance map of the SC region shown in Fig. 2a. No indication of charge order is present in the SC region.

3. The imaging of CO phase boundary

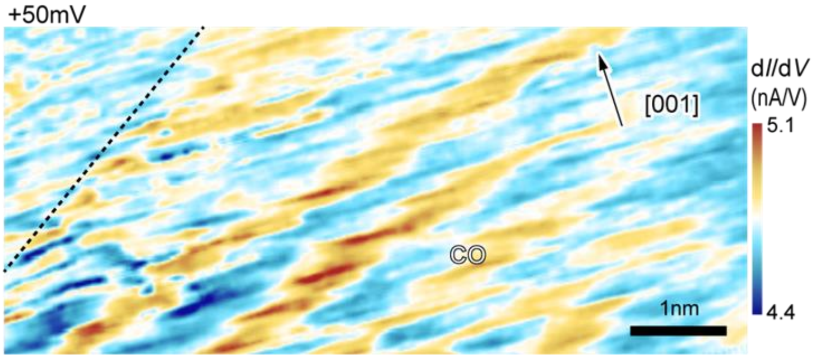

Supplementary Fig. 3: Differential conductance map of a different location at a CO phase boundary taken at +50 mV (200 pA). The appearance of the boundary is comparable to that of Fig. 4a, albeit the conductivity map is noisier at the lower tunneling voltage used here.

4. The d*I*/d*V* spectra on Cu-O terminated surface

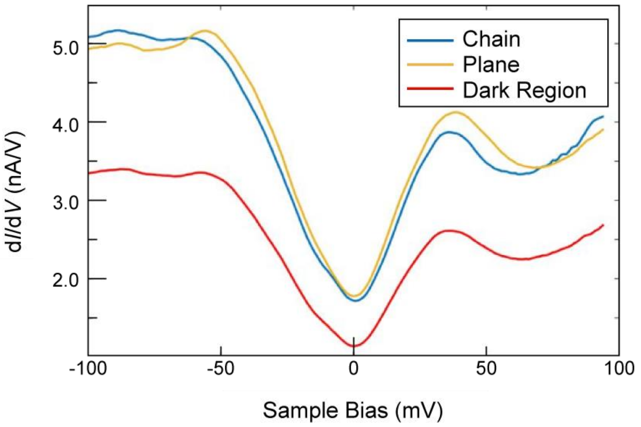

Supplementary Fig. 4: The d*I*/d*V* spectra acquired on chain (blue) and plane layers (yellow) compared to the dark region in between (red). The spectra were normalized to constant tip-sample separation due to the height difference of 0.4 Å between and on top of the atomic rows, assuming a typical decay constant of 1 $Å^{-1}$.

Supplementary Fig. 4 shows the spectroscopic data in the dark region between the CuO/CuO_2 layers (red line). In order to compare it with the spectroscopy on top of the CuO/CuO_2 layers, we need to take into account that the tip approaches the surface in the dark region. Using the height difference between the dark region and the atomic rows and a typical decay constant of the DOS of 1 $Å^{-1}$ we normalized the spectroscopy in first approximation to identical tip-sample separation. The result is given in Supplementary Fig. 4. The conductance (and thus DOS) in the dark region is considerably lower than on top of CuO/CuO_2 layers. The overall shape is very similar. This suggests that the LDOS localized at CuO/CuO_2 layers decays along the **c** direction, but with significant overlap between adjacent CuO/CuO_2 layers.
